# Bionomic aspects of *Lutzomyia evansi* and *Lutzomyia longipalpis*, proven vectors of *Leishmania infantum* in an endemic area of non-ulcerative cutaneous leishmaniasis in Honduras

**DOI:** 10.1186/s13071-017-2605-7

**Published:** 2018-01-05

**Authors:** Ángel Mejía, Gabriela Matamoros, Gustavo Fontecha, Wilfredo Sosa-Ochoa

**Affiliations:** 1 Microbiology School, Universidad Nacional Autónoma de Honduras, Tegucigalpa, Honduras; 2 Microbiology Research Institute, Universidad Nacional Autónoma de Honduras, Tegucigalpa, Honduras

**Keywords:** Non-ulcerative cutaneous leishmaniasis, Visceral leishmaniasis, Barcoding

## Abstract

**Background:**

Some *Lutzomyia* species are the vectors of human leishmaniasis in the Americas. Visceral and cutaneous leishmaniasis are both endemic in the Pacific region of Honduras, but the non-ulcerative form is the more frequent clinical manifestation in this region, where *Lutzomyia longipalpis* is the most abundant and the only incriminated vector. Taxonomic identification and distribution studies of sand flies are important to understand the epidemiology and to control these neglected tropical diseases.

**Results:**

Here, we identified more than 13,000 *Lutzomyia* specimens captured in Isla del Tigre, Honduras, through a classical morphological approach. The two most common species were *Lutzomyia evansi* and *Lu. longipalpis*, and this is the first report of three *Lutzomyia* species on this island. The blood meal source was successfully identified for five sand fly species. A barcode analysis using the *cox*1 mitochondrial marker proved to be effective in discriminating between species and seems to be a valuable tool for future epidemiological studies including a wider geographical area.

**Conclusion:**

This study updates the diversity and blood meal sources of *Lutzomyia* species in an island endemic for non-ulcerative cutaneous leishmaniasis in the Pacific region of Honduras, and determines the effectiveness of the barcoding approach to discriminate species, as a complementary tool to classical morphology.

## Background

Leishmaniasis is a complex of human and zoonotic diseases caused by parasites of the genus *Leishmania*. In the American continent *Leishmania* parasites are transmitted to their hosts through the bite of hematophagous insects of the genus *Lutzomyia* [1]. To date, leishmaniases are considered as one of the main “neglected tropical diseases” in the world and are a major obstacle for the development of countries like Honduras because of its strong association with poverty and healthy life years lost from disability [2–4].

According to the National Surveillance Laboratory and the Panamerican Health Organization Office in Honduras, the number of human infections caused by *Leishmania* parasites in Honduras during 2015 was 2054, mostly as cutaneous presentations [5] (Table 1).

**Table 1:** Number (*n*) of *Leishmania* infections reported in: Honduras (HN), the Pacific region of the country comprising Choluteca and Valle departments (PR), and in Isla del Tigre (IT), from 2009 to 2015. The four clinical forms are reported separately

	CL			NUCL			MCL			VL		
	IT *n* (%)	PR *n* (%)	HN	IT *n* (%)	PR *n* (%)	HN	IT *n* (%)	PR *n* (%)	HN	IT *n* (%)	PR *n* (%)	HN
2009	0	23 (4.4)	525	43 (4.5)	771 (81.5)	946	0	0	4	0	2 (100)	2
2010	0	5 (0.6)	771	18 (3.6)	491 (98.6)	498	0	0	3	0	9 (100)	9
2011	0	0	1546	22 (5.9)	260 (70.0)	371	0	0	13	0	5 (83.3)	6
2012	12 (1.14)	18 (1.7)	1056	95 (10.8)	673 (76.7)	877	0	0	2	0	0	0
2013	0	1	1316	93 (12.2)	491 (64.6)	760	0	0	3	0	3 (100)	3
2014	3 (0.32)	3 (0.3)	935	214 (22.5)	778 (81.9)	950	0	0	14	1 (50.0)	2 (100)	2
2015	0	0	1039	89 (9.1)	723 (73.6)	982	0	0	27	0	5 (83.3)	6

*Abbreviations: CL* cutaneous, *NUCL* non-ulcerative, *MCL* muco-cutaneous, *VL* visceral

In Honduras there are four known manifestations of human leishmaniasis, and they are classified according to clinical signs, geographical distribution, parasite species, and vector species in each area. Non-ulcerative cutaneous leishmaniasis (NUCL, also called atypical cutaneous leishmaniasis [6]), cutaneous (CL) and visceral leishmaniasis (VL) are endemic in Southern Honduras (Pacific Region) due to specific eco-epidemiological characteristics [7, 8]. The ulcerative disease contributed to 50.6% of total national cases in 2015, while the non-ulcerative cutaneous form represented 47.8% of the parasite infections. The Pacific region of Honduras (including Choluteca and Valle departments or provinces) reported 723 (73.6%) cases of NUCL in 2015, and Isla del Tigre alone (belonging to Valle department) contributed with 89 (9%) of the total amount of cases. The parasite responsible for both clinical manifestations (VL and NUCL) seems to be *Leishmania infantum* (syn. *L. chagasi*) [6, 8].

In the Neotropics there are nearly 500 species of *Lutzomyia* identified according to morphological characters [9], and at least 30 of which have been described as *Leishmania* vectors [10]. In Honduras, 31 *Lutzomyia* species have been reported and 12 show anthropophilic behavior [11]. *Lutzomyia longipalpis* is considered vector of only *Leishmania* (*L.*) *infantum* and not of other parasite species. Although *Leishmania* species causing cutaneous leishmaniases have been identified by molecular methods in *Lu. longipalpis*, those findings have no epidemiological significance [12]. *Lutzomyia longipalpis* is also the best described species in Honduras and is the only one from which *L. infantum* strains have been isolated. For these reasons *Lu. longipalpis* is considered as the main vector of VL in southern Honduras [4, 5]. However, other sand fly species have been highlighted as permissive vectors of the parasite in the Americas such as *Lu. evansi*, *Lu. fischeri* and *Lu. migonei*, among others [13, 14].

On the other hand, studies on the feeding habits of *Leishmania* vectors is as important as the description of the circulating species in a geographical area, because that knowledge contributes to identifying potential reservoirs, to understanding their role in the maintenance of insect populations in a locality, levels of anthropophilia, as well as in defining the zoonotic or anthroponotic cycles of the disease [15, 16].

*Lutzomyia* specimens are classically identified upon morphological internal characters such as spermatheca and genitalia, among other structures, although there are several disadvantages with this approach. For example, this method requires a vast experience and technical training and is laborious and time-consuming; also it is not always possible to have good quality specimens because of damages during capture, transport or mounting [17]. In addition, some populations of sand flies show some degree of phenotypic plasticity [18], or cryptic species may be co-existing in the same location [19, 20]. The development of molecular tools based on DNA sequences, allows to complement taxonomic identification based solely on morphology. One of the most commonly used markers for the identification of sand flies is the gene cytochrome *c* oxidase subunit 1 (*cox*1), because of its high level of conservation [21, 22]. Therefore, the aim of the present study was to identify the species of *Lutzomyia* on a Mesoamerican Pacific island where leishmaniasis is endemic, using morphological and molecular methods, as well as to determine the blood meal source of these sand flies.

## Methods

### Area of study and collection of phlebotomine sand flies

**Fig. 1:**
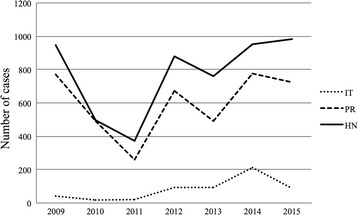
Number of human leishmaniasis cases in Honduras (HN), the Pacific Region of Honduras (PR), and Isla del Tigre (IT), from 2009 to 2015

**Fig. 2:**
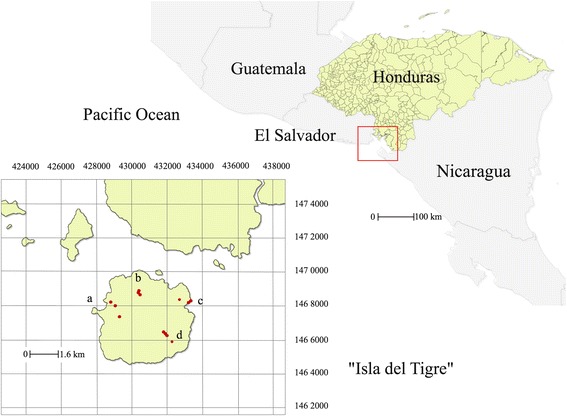
Location of sites where *Lutzomyia* specimens were collected in Isla del Tigre, Gulf of Fonseca, Honduras: **a**, Caracol; **b**, Ceibita; **c**, Las Pelonas; **d**, Islitas

The capture of phlebotomine sand flies was carried out in 4 villages (Ceibita, Caracol, Islitas, and Las Pelonas) of an island called Isla del Tigre, in the Pacific region of Honduras. This island was selected for this study due to the endemicity of human leishmaniasis, registering 214/950 (22.5%) of national NUCL cases in 2014, and 89/982 (9.1%) in 2015 (Fig. 1 and Table 1). The island is located in the Gulf of Fonseca (13.2°N and 87.6°W), and has a maximum height of 783 m above sea level (masl) (Fig. 2). Insects were captured using CDC light traps without chemical attractant [23] in 13 collection points between August 2012 and March 2013 during one week of each month. Traps were installed in an extra-, intra- and peri-domiciliary fashion.

The first collection points were houses with the presence of domestic animals. Two traps were installed there (one intra-domiciliary and one peri-domiciliary). Peri-domiciliary traps were placed at sites of rest from domestic animals or next to the latrines. The traps were placed at a minimum distance of 25 masl. Between them in the mountain, located at the center of the island. Three traps were placed in extra-domiciliary environments, with little or no anthropogenic intervention (crops, mountain trails, etc.). The first trap was placed 100 m away from the peri-domicile, and the second and third traps were placed at 100 m away from each other.

Ceibita village (3 collection points) is located at the northern end of the island, and was the village with the largest number of houses. Its main economic activities are subsistence agriculture and animal breeding. Two of three points were houses, and the third point (with the higher altitude) was placed in a semi-wooded environment near a field of corn of moderate extension. Caracol village (3 collection points) is located at the northwestern end of the island. Its main activities are fishing, animal breeding, and subsistence agriculture with small family gardens. Two collection sites included one house each, and the third point (extra-domiciliary) at a higher altitude was placed in a pigsty that supplies pork products to the local population. Islitas village (4 collection points) is located at the southeastern end of the island. This village has fewer houses, and most of them were located on the side of the road that surrounds the island. Its population is dedicated to animal breeding, hunting wild animals, and timber extraction. The first point evaluated at this location was a house, and the remaining 3 points were extra-domiciliary into the wild up in the mountain environment. Las Pelonas village (3 collection points) is located at the northeastern end of the island. Economic activities focus on fishing, agriculture and breeding of pigs, poultry and cattle on a small scale. Lands are flatter and closer to the coast compared to the other 3 communities.

Collected insects were preserved in absolute ethanol and transported to the laboratory in Tegucigalpa, the capital city. Specimens of the genus *Lutzomyia* were separated from other insects. Females and males were classified through visualization of genital structures [11].

### Taxonomic identification of *Lutzomyia* species

Ethanol-preserved specimens were hydrated in 1× PBS for 30 min and then cleared with 10% KOH for 2 h and 1× PBS for 30 min. Subsequently, the cleared specimens were mounted in permanent microscope slides with Hoyer’s medium. Identification of specimens was based on taxonomic morphology of ascoids, wing venation, thorax coloration, the spermathecae and cibarium of the females, and the genitalia of males [11].

### Identification of blood meal sources

DNA extraction was carried out from females of the genus *Lutzomyia* engorged with blood (with or without eggs). Specimens were dissected, identified, and pooled with other 3 individuals of the same species from the same capture site in vials with 25 μl of 5% Chelex® 100 (Bio-Rad Lab Inc., Hercules, California, USA).

Sand fly pools were macerated and vortexed for 20 s, followed by a brief centrifugation and incubation at 97 °C for 30 min. The pools were centrifuged at 13,000× *rpm* for 10 min, and the supernatant was transferred to sterile vials and stored at -20 °C [24].

In order to identify the source of the blood intake of the sand flies, four specific PCR reactions were separately performed for dog, chicken, pig, and human, as described by Pizarro et al. [15]. Short interspersed nuclear elements (SINEs) were the target for non-human species, while an Alu element-based; a long interspersed element (LINE) was amplified for detection of human DNA.

### Imaging and tissue lysis for barcode analysis

Twenty-one specimens belonging to seven species of the genus *Lutzomyia* were processed according to the DNA barcoding workflow. This procedure includes imaging, tissue sub-sampling, tissue lysis, DNA extraction, PCR amplification and sequencing of the *cox*1 marker. Specimens were imaged using the Leica Application Suite (LAS). These 21 ethanol preserved insects were analyzed for DNA barcoding in 2016 and were previously identified by conventional microscopic methods during 2013. The images used for barcoding purposes were not intended to identify the insects.

Due to the small size of the specimens, sub-sampling was not performed; instead, the entire organism was processed into plate-wells containing 30 μl of 95% ethanol. Plates were centrifuged at 1000×*g* for 30 s and incubated for 2 h at 56 °C in order to evaporate the ethanol. For each well, 50 μl of lysis buffer (100 mM NaCl, 50 mM Tris-HCl, pH 8.0, 10 mM EDTA, pH 8.0 and 0.5% SDS, and Proteinase K) were added. The plates were incubated at 56 °C overnight.

### DNA extraction

One hundred microliters of the previous binding mix were added to each well. One hundred and eighty microliters of lysate were transferred into the wells of a glass fiber plate (Pall corp., NY, USA), placed on top of a clean square-well block for binding and washing steps. The plate assemble was centrifuged at 5000× *g* for 5 min in order to bind DNA to the glass fiber membrane. Two washing steps were performed, using 180 μl of protein wash buffer (binding buffer and 96% ethanol), and 750 μl of wash buffer (96% ethanol, 50 mM NaCl, 10 mM Tris-HCl pH 7.4, 50 mM EDTA pH 8.0). The plate was air-dried and stored at 56 °C for 30 min. Forty microlitres of a warmed elution buffer (10 mM Tris-HCL, pH 8.0) were dispensed directly into the membrane in each well of the glass fiber plate and incubated at room temperature for 1 min. The plate was assembled with a DNA Eppendorf plate and centrifuged at 5000× *g* for 5 min to collect the DNA. DNA was stored at 4 °C until further use.

### PCR amplification and sequencing

The PCR reaction mix included 10% trehalose, ddH_2_O, 10× PCR buffer for Platinum Taq DNA polymerase (Invitrogen, Carlsbad, California, USA), 50 mM MgCl_2_, 10 μM of each primer: ZplankF1t1 (5′-TGT AAA ACG ACG GCC AGT TCT ASW AAT CAT AAR GAT ATT GG-3′), ZplankR1t1 (5′-CAG GAA ACA GCT ATG ACT TCA GGR TGR CCR AAR AAT CA-3′), 10 mM dNTPs, Platinum Taq polymerase (5 U/μl); 2.5 μl of DNA was added for a total volume of 12.5 μl.

PCR conditions were as follows: an initial step at 94 °C for 1 min followed by 5 cycles of 94 °C for 40 s, 45 °C for 40 s and 72 °C for 1 min. Thereafter, 35 cycles of 94 °C and 51 °C for 40 s and 72 °C for 1 min and a final extension of 72 °C for 5 min were run. Subsequently, an E-gel 2% agarose (Invitrogen) was performed to confirm the amplification. PCR products were diluted in 25 μl of ddH_2_O for clean-up with magnetic beads. Cycle sequencing was performed by adding 5 μl of 10% trehalose, 2 μl of Big Dye®, 1.87 μl of 5× sequencing buffer (400 mM Tris-HCl pH 9.0, 10 mM MgCl_2_, 0.87 μl of ddH_2_O, 1 μl of 10 μM of each primer ZplankF1t1/ZplankR1t1 and 1.2 μl of PCR product. The sequencing program was: 1 cycle at 96 °C for 2 min, 30 cycles of 96 °C for 30 s, 55 °C for 15 s and 60 °C for 4 min. Subsequently the plates were submitted for sequencing with M13F and M13R primers to the Canadian Centre for DNA Barcoding (CCDB).

Sequences were edited using the CodonCode software (CodonCode Corp., Centerville, MA, USA) and compared against the DNA barcode library (http://www.boldsystems.org) to infer specimens’ identification. The higher match, sequences overlap (nt), and match process ID was recorded.

A neighbor-joining tree was built through the Geneious® v.9.1.7 software based on 13 sequences of local specimens and 137 sequences from the BOLD system representing 74 *Lutzomyia* species. All individuals were aligned using the Geneious alignment software including a 529 bp fragment of the *cox*1 gene. Distances were computed using the Tamura-Nei model and Bootstrap values from 10,000 replicates. The percentage of identical sites and the pairwise percentage identity was calculated within and between species.

## Results

### Identification of *Lutzomyia* species

**Fig. 3:**
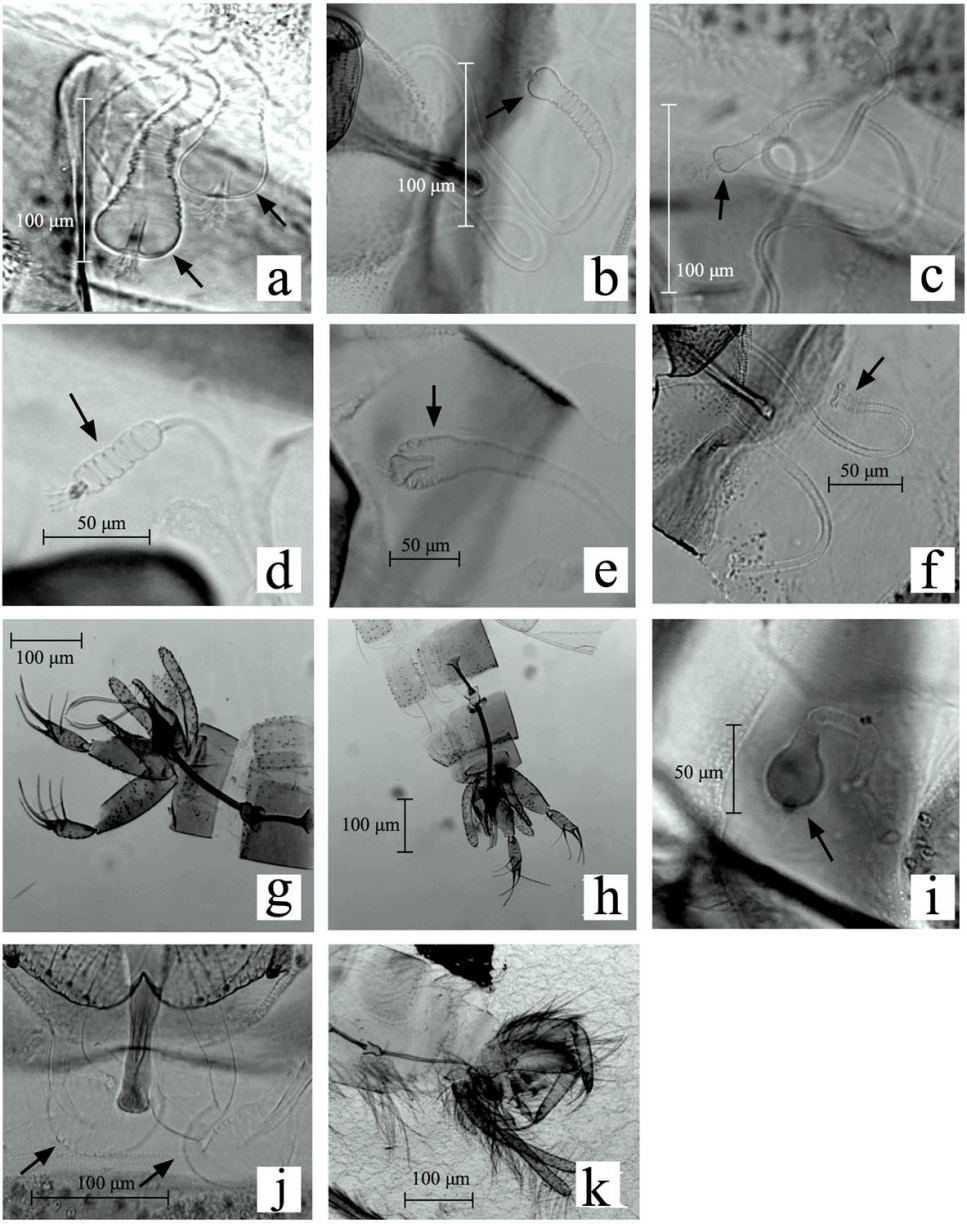
Genital structures of *Lutzomyia* specimens from Isla del Tigre, Honduras. **a** *Lu. chiapanensis* (female). **b** *Lu. cruciata* (female). **c** *Lu. gomezi* (female). **d** *Lu. longipalpis* (female). **e** *Lu. evansi* (female). **f** *Lu. cruciata* (female). **g** *Lu. sanguinaria* (male). **h** *Lu. trapidoi* (male). **i** *Lu. cayennensis* (female). **j** *Lu. zeledoni* (female). **k** *Lu. panamensis* (male)

A total of 13,248 specimens belonging to the genus *Lutzomyia* were collected. Seventy-eight percent were female. Ten species were identified through morphological characters (Fig. 3). Sixty-four (4.83%) specimens could not be identified due to deterioration during storage and transport and were recorded as *Lutzomyia* spp. The most abundant species was *Lu. evansi* followed by *Lu. longipalpis* (Table 2)*.* This is the first report of three *Lutzomyia* species in Isla del Tigre (*Lu. evansi*, *Lu. cayennensis* and *Lu. panamensis*). *Lutzomyia cayennensis cayennensis* is the only subspecies reported in Honduras for the *Lu. cayennensis* complex [11]; however we do not have enough evidence to demonstrate that our specimens belong to this subspecies.

**Table 2:** Number of *Lutzomyia* spp. specimens captured in Isla del Tigre, Honduras, classified according to sex and location

*Lutzomyia* spp./Location	Islitas		Ceibita		Caracol		Las Pelonas		Total (%)
	M	F	M	F	M	F	M	F	
*Lu. evansi*	107	261	460	7933	194	636	50	139	9780 (73.82)
*Lu. longipalpis*	52	20	807	508	151	43	593	97	2271 (17.14)
*Lu. gomezi*	6	23	8	33	50	151	8	70	349 (2.63)
*Lu. cruciata*	26	37	40	41	55	117	11	25	350 (2.64)
*Lu. chiapanensis*	3	10	2	22	4	24	32	92	189 (1.42)
*Lu. sanguinaria*	2	0	22	0	32	0	116	0	172 (1.29)
*Lu. zeledoni*	2	15	1	16	2	24	0	8	68 (0.51)
*Lu. cayennensis*	0	1	0	0	0	0	0	2	3 (0.02)
*Lu. trapidoi*	0	0	1	0	0	0	0	0	1 (0.00)
*Lu. panamensis*	1	0	0	0	0	0	0	0	1 (0.0)
*Lutzomyia* sp.	4	1	16	23	4	6	7	3	64 (4.83)
Subtotal	203	368	1357	8576	492	1001	817	434	
Total *n* (%)	571 (4.31)		9933 (74.97)		1493 (11.26)		1251 (9.44)		13,248 (100.00)

*Abbreviations: F* female, *M* male

According to the location of the traps, the extra-domiciliary ecotope showed a higher capture rate of sand flies (79.66%), followed by the peri-domiciliary (12.34%) and the intra-domiciliary ecotopes (8%). The most frequent species in the extra-domiciliary ecotope was *Lu. evansi*, while *Lu. longipalpis* was most frequent in the peri-domicile setting (data not shown). Although the analysis of species diversity reveals that Islitas, is the location with greater specific richness, the four localities are very similar to each other in terms of species composition, which defines them as relatively homogeneous communities. In terms of the number of collected specimens, Ceibita contributed nearly 75% of all individuals, and 86% of *Lu. evansi* specimens. The extra-domiciliary ecotope showed most of individuals of *Lu. evansi* [*n* = 8779 (89.8%)], while the intra-, and the peri-domicile revealed 414 (4.2%) and 587 (6%), respectively.

### Identification of blood meal sources

We found that six species harbored blood and it was possible to identify the blood meal source for five species (*Lu. longipalpis*, *Lu. cruciata*, *Lu. evansi*, *Lu. gomezi* and *Lu. chiapanensis*) (Table 3). All exhibited a zoophilic feeding behavior involving at least the use of one animal as a food source. The most frequent food source was pig (*Sus scrofa*), followed by dog (*Canis familiaris*), chicken (*Gallus gallus*), and human (*Homo sapiens*). *Lu. longipalpis* and *Lu. cruciata* were the only two species with anthropophilic behavior. It was not possible to determine the blood meal source of *Lu. zeledoni*. The total of the tested specimens were fed on a single animal source. No mixed blood meals were detected.

**Table 3:** Blood meal of *Lutzomyia* species

*Lutzomyia* spp.	*Homo sapiens*	*Canis familiaris*	*Sus scrofa*	*Gallus gallus*	Total *n* (%)
*Lu. longipalpis*	1	1	8	1	11 (14.47)
*Lu. gomezi*	–	–	7	–	7 (9.21)
*Lu. cruciata*	1	1	–	–	2 (2.63)
*Lu. chiapanensis*	–	–	1	–	1 (1.31)
*Lu. evansi*	–	15	32	8	55 (72.36)
*Lu. zeledoni*	–	–	–	–	–
Total (%)	2 (2.63)	17 (22.36)	48 (63.15)	9 (11.84)	76 (100)

### Barcode analysis

A fragment of the mitochondrial *cox*1 gene was sequenced from 21 specimens morphologically classified into 7 *Lutzomyia* species. Only a few specimens were selected for molecular analysis because most of the insects were permanently mounted in Hoyer’s medium.

The *cox*1 sequence length was 622 bp by direct sequencing. Barcoding could not be performed to three of eleven species (*Lu. sanguinaria*, *Lu. trapidoi* and *Lu. panamensis*) because all the individuals from these species were permanently mounted for taxonomic identification four years ago. Only 13 out of 21 specimens were sequenced with enough quality to allow subsequent analysis (Table 4). Each species was analyzed using 1–3 specimens. Sequences from the 13 collected specimens showed an average A + T bias (66.7%) relative to the C + G content. Sequences and trace files are available in the BOLD project named: “Identification of *Lutzomyia* sp. recovered at Amapala Honduras [HNLUZ]”.

**Table 4:** Comparison of the species-level identifications of collected *Lutzomyia* specimens with the identifications as determined by DNA barcoding

Phenotypic identification	BOLD Sample ID	BOLD higher match	% Highest match	Overlap (nt)	Match process ID
*Lu. longipalpis*	HNLUZ004-17	*Lu. longipalpis*	99.03	609	MEXSM003-12
*Lu. longipalpis*	HNLUZ005-17	*Lu. longipalpis*	98.71	609	MEXSM003-12
*Lu. evansi*	HNLUZ001-17	*Lu. evansi*	93.98	594	GBMIN23074-13
*Lu. gomezi*	HNLUZ008-17	*Lu. gomezi*	99.03	558	GBPSY020-14
*Lu. gomezi*	HNLUZ010-17	*Lu. gomezi*	99.19	561	GBPSY020-14
*Lu. gomezi*	HNLUZ012-17	*Lu. gomezi*	99.03	558	GBPSY020-14
*Lu. cruciata*	HNLUZ014-17	*Lutzomyia* sp.	88.83	571	None
*Lu. zeledoni*	HNLUZ019-17	*Psychodidae*	98.29	609	None
*Lu. zeledoni*	HNLUZ020-17	*Psychodidae*	98.29	609	None
*Lu. zeledoni*	HNLUZ022-17	*Psychodidae*	98.10	609	None
*Lu. chiapanensis*	HNLUZ015-17	*Psychodidae*	91.36	609	None
*Lu. cayennensis*	HNLUZ024-17	*Phytoliriomyza melampyga*	91.10	609	None

The DNA barcode analysis enabled us to correctly identify 3 *Lutzomyia* species (*Lu. longipalpis*, *Lu. evansi* and *Lu. gomezi*) as determined from the morphological identifications (Table 4). Sequences contained an average of 66.7% of A + T pairs for all codons. The remaining 4 species (*Lu. cruciata*, *Lu. zeledoni*, *Lu. chiapanensis*, and *Lu. cayennensis*) showed low percentage matches with specimens of the family Psychodidae or with some unidentified species of the genus *Lutzomyia.*

The mean interspecific nucleotide divergence was 28.4% with a pairwise percentage identity of 88.1% for a 529 bp sequence. The intraspecific nucleotide divergence ranged from 0.2% (*Lu. zeledoni*) to 0.6% (*Lu. gomezi*), with a pairwise percentage identity of 99.6–99.9%.

**Fig. 4:**
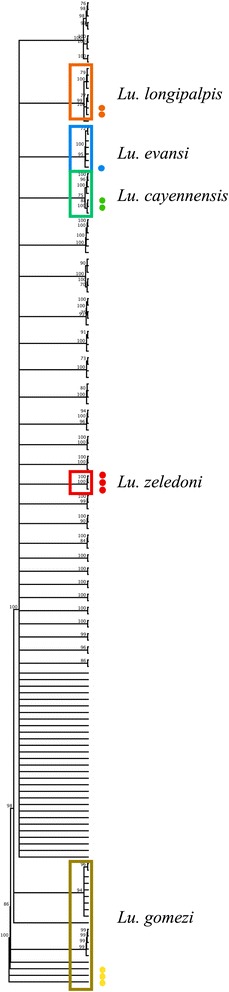
Neighbor-joining tree inferred from the *cox*1 gene of *Lutzomyia* spp. Bootstrap values from 10,000 replicates are shown. The distances were computed using the Tamura-Nei model. Isolates from this study are shown with color dots

When the sequences obtained in this study were analyzed along with 150 BINs available in the BOLD system for the genus *Lutzomyia*, the NJ tree revealed that *Lu. evansi*, *Lu. longipalpis,* and *Lu. gomezi* grouped with specimens of these species (Fig. 4); *Lu. cayennensis* formed a distinct but neighbour clade to other *Lu. cayennensis* specimens. However, *Lu. zeledoni* and *Lu. cruciata* did not group with any other specimens of these two species. Two different species (*Lu. chiapanensis* and *Lu. cayennensis*) grouped closely in a single branch due to a high interspecific pairwise identity (99.2%). The dendrogram also shows two clearly differentiated clades for *Lu. longipalpis*. The first clade includes 6 specimens (BINs: GBPSY305-14–GBPSY308-14, MEXSM002-12–MEXSM003-12) isolated from Colombia and Mexico, together with the two specimens of Honduras, while the second clade included four other specimens from Colombia (BINs: GBMIN23018, -23019, -23051, -23052).

## Discussion

This study investigated the diversity of species of the genus *Lutzomyia*, vector of leishmaniasis in the Americas, on a highly endemic island for non-ulcerative cutaneous leishmaniasis (NUCL). During the decade of the 1990s some investigations were carried out in this geographical region of Honduras which (i) incriminated *Lu. longipalpis* as the vector of *Leishmania infantum* [7, 8]; (ii) demonstrated the predominance of *Lu. longipalpis* in the island; and (iii) described its behaviour [25]. Our findings were intended to provide an update on the current distribution of *Leishmania* vectors in the Pacific region of Honduras, plus the detection of its blood meal sources, and the use of a barcoding approach for species identification.

A large number of specimens of the genus *Lutzomyia* were collected and classified into ten species. *Lutzomyia longipalpis* has historically been considered the most common and relevant species in *Leishmania* transmission in the Pacific region of Honduras [25], and is the only species in which natural infections have been reported [7, 8].

This finding is consistent with Raymond et al. [26] who reported *Lu. longipalpis* and *Lu. evansi* as the two most common species in the Pacific region of Nicaragua, with similar ecological characteristics as those of the Honduran Pacific. There are at least three reasons that could justify the predominance of *Lu. evansi* in this study: (i) natural changes in the population dynamics of the insect; (ii) the longer capture time of this study (9 months) when compared to captures made over a few days in previous reports; and (iii) the procedure to collect sand flies also in the extra-domicile instead of the peri-domicile (edge effect) [25]. Although natural infections of the parasite in *Lu. evansi* have not been demonstrated in Honduras, its potential role in the transmission of leishmaniasis cannot be ruled out, as reported for other American countries [13]. As a consequence, it would be interesting to carry out further studies to demonstrate the presence of the parasite in this phlebotomine. Taking into consideration the extra-domiciliary predominance shown by *Lu. evansi*, in contrast to the peri-domiciliary behavior of *Lu. longipalpis*, distinct transmission cycles of *L. infantum* for each species could be proposed. For example, *Leishmania* infections occurring in the agricultural working areas could be attributed to *Lu. evansi*, while infections around the houses could be mostly produced by *Lu. longipalpis.* However, these hypotheses will require further studies. A second hypothesis suggests that *Lu*. *evansi* could be maintaining the sylvatic cycle of the disease, and this could be also proven in the future through the blood identification of sylvatic animals in this sand fly species.

This is also the first report of one *Lutzomyia* species in Isla del Tigre which has not been incriminated in the transmission of the parasite (*Lu. cayennensis*)*.* Two other species observed here were *Lu. gomezi* and *Lu. panamensis*, vectors of *L. braziliensis* and *L. panamensis* in the Americas [27, 28], but this would have no epidemiological relevance due to lack of transmission of these parasites species in the Pacific region of Honduras, although they may transmit other *Leishmania* species.

Despite the low dispersal abilities of *Lutzomyia* (e.g. 500 m for *Lu. longipalpis*) [29], the observed homogeneity in the distribution of the sand fly species in the island could be due to the small size of the territory (75.2 km^2^) and to the absence of natural or climatic barriers that could structure fragmented populations. Perhaps expanding the area of ​​study to continental soil could evidence some level of population structure in *Lutzomyia* species. Despite this relative species homogeneity, the 13 collection points showed some interesting differences in the number of collected specimens.

Notoriously, Ceibita was the village with the highest number of sand flies collected (75%) (Table 2). Perhaps the more intensive human intervention of the soil and a more abundant agricultural work are responsible for creating the conditions for the development of the life-cycle of those insects (e.g. soil modification, irrigation of crops with presence of moisture even in dry seasons, the presence of domestic and wild animals seeking food in crop fields, the presence of organic matter, shading of backyards, debris and other factors). A remarkable fact is that 86% of the catches of *Lu. evansi* occurred at the extra domiciliary point of collection at Ceibita. This large population caught could be attributed to an edge effect in this area, where humans are continuously penetrating the forest.

Caracol was the second village with the highest numbers of collected sand flies, and the extra-domicile was the largest contributor of the 3 collection sites. The large number of pigs in the area could be the main explanation for this result. 63.15% (Table 3) of the engorged blood belonged to pigs, which is consistent with this hypothesis and suggests that this mammal may be a key element for the maintenance of the insect populations in extra- and peri-domiciliary ecotopes. Las Pelonas occupied the third place regarding the number of specimens collected. The highest number of insects was obtained in the peri-domicile of a house located next to the beach, with flat and sandy soils, and close to a small piggery and a chicken coop. Likewise, the presence of domestic animals such as pigs, chickens and dogs would allow the presence of the insects in peri-domiciliary and intra-domiciliary environments. Traps located at Islitas revealed the lowest number of specimens. Fewer animals around the houses could have reduced the food sources available for the sand flies in comparison with the other three villages, with a greater presence of pets and wild animals.

This study also investigated the blood meal sources of the sand flies from Isla del Tigre. Only *Lu. longipalpis* and *Lu. cruciata* showed an anthropophilic behavior. This finding is in agreement with previous reports of authors from Brazil and Mexico [30–32] and reaffirms the potential role of *Lu. longipalpis* in the transmission of visceral leishmaniasis. Five out of six *Lutzomyia* species revealed to be engorged with animal blood. The most frequent source of blood meal was pig, followed by dog.

Due to cultural habits of animal breeding in this region, pigs live very close to the humans and their houses. This habit is relevant in the context of this study, since *Lu. evansi* have been proven to feed on pigs [33]. For this reason these animals could be considered as potential reservoirs of the parasite. The dog has been defined as the main reservoir of *L. infantum*, both in Honduras and in the Americas [7, 34–36], therefore our finding supports its leading role in establishing the domiciliary cycle of leishmaniasis on the island. Although chickens are refractory to *Leishmania* infections, their presence in the peridomicile is undoubtedly a risk factor that favors the presence and maintenance of sand flies in the human habitat [37, 38]. With respect to the large population of *Lu. evansi* captured in Ceibita, it would be interesting to conduct further studies of other potential food sources among sylvatic animals, such as *Didelphis marsupalis* and small rodents [39, 40]. The source of the blood meal of *Lu. zeledoni* was not identified, suggesting a different food source to the four analyzed in this study, such as armadillos [41], horses, rats, cats [42], or cows [43], among others.

Thirteen specimens belonging to seven morphologically identified *Lutzomyia* species were DNA barcoded in this study. This approach proved to be useful for correctly identifying three *Lutzomyia* species. However, four of seven species could not be identified based only on genetic divergences when their sequences were queried against public databases of the BOLD system or NCBI. This could be due to the lack of BINs or accession numbers for these species in the BOLD system and the NCBI databases, respectively. This result confirms that *cox*1 is a useful molecular marker to identify species when there are enough records in the databases [17, 44, 45], but when there are only a few sequences available, the use of other molecular markers such as ribosomal ITS spacers or *nad*1 (nicotinamide adenine dinucleotide dehydrogenase 1), together with conventional taxonomy remains fundamental to report new species within a geographical region [19, 46, 47].

Sequences from the 13 collected specimens showed an average A + T bias (66.7%) relative to the C + G content similar to the expected range for sand flies [48, 49]. The variability between species (12–18%) was similar to that reported in other studies conducted with sand flies from the Americas. Nzelu et al. [17] reported a range from 8.39 to 19.08% when 19 species from Peru were analyzed, and Contreras et al. [48] found a mean variation of 19% among 26 species collected from Colombia. However, intraspecific variability was less than 1%, which can be attributed to the low number of specimens sequenced from each species in this study (max. 3). Nevertheless it seems there is no overlap between inter- and intraspecific divergences supporting the barcoding gap that allows to assign taxonomic status to our specimens [50].

The neighbor-joining tree showed distinctively clustered sequences from local specimens of *Lu. longipalpis* and *Lu. evansi* together with specimens of the same species from other regions of the continent (Fig. 4). However, *Lu. longipalpis* seemed to be grouped into two different clusters. The first cluster included the specimens from Honduras, four sequences from Colombia and two from Mexico, and the second cluster comprised four specimens from Colombia. This separation in two haplotypes will require further studies with more specimens collected from more localities in the Mesoamerican region in order to assess the existence of more than one genetic population or even cryptic species within the *Lu. longipalpis* complex, as suggested by other authors from Brazil [51–53]. The relationships visualized in the dendrogram between sequences from this study of *Lu. gomezi* and accessions from Colombia and Panama indicate that these species are identifiable by this molecular marker. However this is not the case for *Lu. cayennensis*, which clustered with sequences of unidentified species of the genus *Lutzomyia* from Colombia, and are separated of other sequences of *Lu. cayennensis*. This may be due to the diversity of the *cayennensis* complex including eight subspecies [27]. The sequences of *Lu*. *zeledoni*, *Lu. cruciata* and *Lu. chiapanensis* did not cluster with any accession due to lack of previous records in the databases.

## Conclusions

In conclusion, our findings updated the diversity of *Lutzomyia* species in an island endemic for human leishmaniasis in the Pacific region of Honduras, and provided information on the blood meal sources of these vectors. This study also showed the effectiveness of the barcoding approach to discriminate species, as a complementary tool to morphology-based identification. Further investigations with a larger number of specimens collected from a wider geographical area would improve the knowledge regarding the distribution of *Leishmania* vectors in Mesoamerica.

## Abbreviations

BIN: barcode Index Number
BOLD: Barcode of Life Data Systems
CCDB: Canadian Centre for DNA Barcoding
*cox*1: cytochrome *c* oxidase subunit 1 gene
LINE: long interspersed element
PCR: polymerase chain reaction
SINEs: short interspersed nuclear elements
